# Internal surface electric charge characterization of mesoporous silica

**DOI:** 10.1038/s41598-018-36487-w

**Published:** 2019-01-15

**Authors:** Tumcan Sen, Murat Barisik

**Affiliations:** Department of Mechanical Engineering, Izmir Institute of Technology, Izmir, 35430 Turkey

## Abstract

Mesoporous silica is an emerging technology to solve problems of existing and to support projected revolutionary applications ranging from targeted drug delivery to artificial kidney. However, one of the major driving mechanisms, electric charging of internal mesoporous surfaces, has not been characterized yet. In the nanoscale confinements of mesoporous structures made of pore throats and pore voids, surface charges diverge from existing theoretical calculations and show local variation due to two occurrences. First, when the size of pore throat becomes comparable with the thickness of ionic layering forming on throats’ surfaces, ionic layers from opposite surfaces overlap so that ionic concentration on the surface becomes different than Boltzmann distribution predicts, and there will no longer be an equilibrium of zero electric potential at pore throat centers. Second, when this non zero potential inside throats becomes different than the potential of pore voids, ionic diffusion from void to throat creates axial ionic variation on surfaces. For such a case, we performed a pore level analysis on mesoporous internal surface charge at various porosities and ionic conditions. Pore parameters strongly affected the average internal charge which we characterized as a function of overlap ratio and porosity, first time in literature. Using this, a phenomenological model was developed as an extension of the existing theory to include nano-effects, to predict the average mesoporous internal surface charge as a function of EDL thickness, pore size and porosity.

## Introduction

Mesoporous silica is a porous material which can be synthesized in a wide range of morphologies (such as spheres, discs and powders) with a pore size ranging between 2 to 50 nm. Mesoporous silica has exceptionally ordered pore structures, high surface area and pore volume, easily tunable-sizes, chemical stability, and biocompatibility. Hence, it provides very promising results across various applications, especially (i) nanoparticle based targeted drug delivery^1^ and bio-imaging^2^, and (ii) nanomembrane based filtration processes^3^ and energy conversion/storage^4^. In these systems, surface silanol groups and the resulting surface charge densities become the determining factor in the corresponding processes. Hence, the design and performance of these applications require an accurate knowledge of the surface charge properties of mesoporous silica at different pore sizes and porosities in various ionic solutions, which has not been fully acquired yet.

The mesoporous systems develop surface charge at both outer and inner surfaces. Existing literature mostly focuses on the outer surface charge of mesoporous nanoparticles^5–7^ or membranes^8–10^ since many of the earlier processes are mostly governed by the outer surface charge. For example, transport of nanoparticles has been controlled by the outer surface charge or desalination processes has been based on the rejection of the certain ions by an opposing outer surface charge of the nanomembrane^11,12^. While practical, controlling of the outer surface charge is not enough for the precise control/design of current existing and future projected applications. For example, drug loading in mesoporous nanoparticles and liquid transport rates through mesoporous membranes are directly related with internal surface charges^13^. Future applications include advanced control of sorption dynamics of vitamins^14^, enzymes^15^, and proteins^16^ for ultrafiltration dialyzers as the artificial kidney^17^; amplified proton activities for fuel-cells^18^ to overcome limited proton conductivity of current polymer membranes; increased ion diffusion for lithium ion pseudocapacitors^19^ to satisfy future energy needs; advanced electrochemical sensors or chemical identifiers *in vivo* and *in vitro* for biosensors^20^. Each require an accurate characterization of the surface properties inside the mesoporous structures for these projected revolutionary applications.

Surface charge density of mesoporous silica characterization has been attempted through experiments. While the most of these studies measured an overall outer surface charge of mesoporous systems, some focused on the internal charge. Generally, two main experimental techniques were used to predict surface charge density inside the mesoporous. The first method involves developing a pressure driven flow and measuring the resulted streaming potential to predict internal surface charge^21–25^, while the second method applies potentiometric titration^26–30^ and measuring adsorption/desorption of protons and ions to estimate internal surface charge. Experiments generally examined the effect of different types pf alkaline chlorides and pH inside the porous system, but a few also focused on the effects of pore shapes^31^, pore size^22,24^, internal surface condition^24^, and porosity^32^. These studies observed the dominant influence of pore network parameters on internal surface charging, such as how a decrease in pore size develops a lower charge inside the membrane system^24^. However, experiments were presented mostly as observations and none of described the underlying physics or correlated observed charging behavior with the properties of mesaporous system. Moreover, both experimental techniques have multiple downsides and difficulties to develop a complete understanding regarding mesoporous surface charging. For instance, the surface charge from streaming potential is calculated based on the Helmholtz-Smoluchowski and the Gouy-Chapman equations, and by ignoring concentration polarization^33^ and electroviscosity effects^34^, which are all invalid for highly overlapped tight mesoporous systems. Second, titration calculations require very long experimental times and additional operations to determine surface area which is very hard to properly estimate. Hence, both methods are extremely sensitive and create unreliable results.

Next option is calculating internal surface charge theoretically. Classically, solution of ionic distribution over a surface (electrical double layer (EDL)) is described by Boltzmann distribution (BD) and electric charge of the corresponding surface is calculated analytically. Most of the existing literature uses charge values based on this theoretical calculation as a function of ionic properties^35,36^. However, such a consideration assumes the surface charge as a material property solely, which is only applicable for planar surfaces sufficiently away from any other bodies. Inside the nanoscale confinements, conduit heights and lengths become comparable with the thickness of EDL such that the ionic distribution on the surface and the resulted surface charge diverge from BD assumptions as a function of system size. Specific for mesoporous systems, there are (i) overlaps between EDLs extending from opposite surfaces in the pore throats, and (ii) overlaps between the ionic distributions of pore voids and pore throats. These two occurrences yield divergence of surface charge from theoretical predictions and local variations, as a function of pore size and porosity, which has been mostly overlooked by the existing literature.

In general, EDL overlap effects on the charge density were studied using Poisson-Boltzmann (PB) or the Poisson-Nernst-Planck (PNP), based on either constant surface charge^37^ or constant wall potential^38^ boundary condition on the surfaces. However, enforcing constant surface charge or potential at the interface does not represent physical response of the surface chemistry. The surface reactions develop based on the local ionic environment at the interface that neither surface charge nor potential remain constant; instead, both of them undergo variation in response to the variation in ionic distribution and create a new equilibrium accordingly. This so-called “Charge Regulation (CR)”^39^ nature of surface chemistry, which is observed by multiple experiments and has been tried to be considered by active surface charge models into numerical calculations as a boundary condition. CR models calculate the effects of the protonation/deprotonation in surface reactions based on the site density of the functional groups. Recently, these models have become very popular in a diverse range of fields such as colloidal science and atomic force measurement (AFM) methods^40,41^. Specifically, studies dedicated to nano-tubes/channels generally focused on flow conditions^42,43^ and assumed infinitely long straight conduits^44,45^. These studies presented strong surface charge variation due to EDL overlap. However, finite size conduits develop ionic variation in the axial direction and disregarding such occurrence yields incorrect estimation of the overall charge density, especially in case of very short conduits; such as mesoporous structures. We just recently addressed this problem in a previous study for straight channels of various lengths and described the electrokinetic development length for surface charging of short nano-channels^46^. Our results showed that the surface charge density deviates from theoretical calculations almost twofold along the entrance and exit regions of short length conduits, in addition to EDL overlap correlated with the defined overlap ratio. Very similar effects develop in mesoporous systems, even more profoundly; but existing theoretical attempts are far from describing the real physical mechanisms as they assume the mesoporous system as a combination of infinitely long surfaces with no EDL overlap^21,22,28,47^. Existing theoretical studies are mostly performed as a curve fitting flat surface CR model parameters to experiments in order to describe the experimentally measured behavior acting different than the flat surface charging. These studies disregarded the real physical mechanisms defining ionic concentrations in a porous system, even though they are well aware that EDL overlaps dominates surface charging^21^. There are some numerical studies dedicated to calculating inner surface charge using extended PB equation with CR, but they either ignored overlap effects^28,48^, only focused on ionic distributions/conductance^49–51^ or solved some specific cases and remain far from providing a general explanation^52^. For such a case, a proper consideration of combined effects of the pore throat EDL overlap and the so-called “pore to throat connectivity” in a mesoporous system as a function of the structural porous parameters is needed to determine the surface charging mechanisms, which does not exist in literature.

In this study, our objective is to characterize the surface charge density of a mesoporous silica system of various pore sizes and porosities for the first time in literature. Local surface charge will be calculated by the previously developed multi-ion charge-regulation model, which considers the effects of the protonation/deprotonation surface reactions, the site density of the functional groups, and pH&salt concentration of the aqueous solution on the silica surface. The Poisson-Nernst-Planck equations will be solved to model ionic diffusion and electrostatic interaction inside mesoporous silica combined with CR model.

## Theoretical and Numerical Background

The ions within the EDL of a semi-infinite charged surface follow the Boltzmann distribution. Combining this ionic distribution with the Poisson equation forms the well-known Poisson-Boltzmann (PB) equation by which the electric potential distribution within the EDL can be resolved. For low wall potential values, PB can further be simplified by applying Debye-Hückel linearization and the resulting equation can be solved analytically. However, this simplification cannot be used for high wall potentials due to the non-linear nature of electric potential near the surface. Even so, non-linear PB can be solved for a known wall potential or surface charge. While some researchers employ either constant wall potential or constant surface charge as a boundary condition to solve PB, most of the recent studies implement CR into PB to include charge regulation nature of surfaces. The latter approach is applicable only if ions obey Boltzmann distribution, which may be invalid for cases where the EDL thickness is comparable with the confinement sizes. Therefore, Poisson-Nernst-Planck (PNP) equations should be utilized for such cases as opposed to commonly used PB, which is the simplified form of PNP.

The current study focuses on a representative volume of a mesoporous silica structure (Supporting Information). Similar with the classical porous system definition, pore size is denoted by the distance between the centers of solid parts (H). The porosity is defined as the ratio of empty pore space to whole pore volume including solids. We kept the aspect ratio of the solid parts as unity. The liquid phase is a symmetric KCl solution that is composed of H^+^, K^+^, Cl^−^ and OH^−^ ions with their bulk values being c_10_, c_20_, c_30_ and c_40_, respectively. For the electroneutrality condition to be satisfied, these bulk concentrations are taken as; *c*_10_ = 10^−*pH*+3^, *c*_40_ = 10^−(14−*pH*)+3^, *c*_20_ = *c*_*kcl*_, *c*_30_ = *c*_*kcl*_ + *c*_10_ − *c*_40_ for pH < 7 and, *c*_20_ = *c*_*kcl*_ + c_10_ − *c*_40_, *c*_30_ = *c*_*kcl*_ for pH > 7. The equations for c_20_ and c_30_ correspond to variation of KOH and HCl, respectively. Resulting EDL can be characterized by debye length $\lambda =1/\kappa =\sqrt{({\varepsilon }_{0}{\varepsilon }_{r}{k}_{B}T)/({N}_{A}{e}^{2}\sum {c}_{i}{{z}_{i}}^{2}})$. Here; ε_0_ and ε_r_ are the permittivity of vacuum and dielectric constant of the aqueous solution, k_B_ is the Boltzmann constant, T is the temperature, N_A_ is the Avogadro constant, e is the elementary charge, *z*_*i*,_ and *c*_*i*_ are the valence and molar concentration of the *i*^th^ ionic species (i = 1 for H^+^; i = 2 for K^+^; i = 3 for Cl^−^; i = 4 for OH^−^). It should be noted that dielectric constant may show variation based on the strength of local electric potential gradient in the EDL, but its effects may be disregarded as against the other variances in the system^53^. Furthermore, the ionic mass transport and electrostatic potential are governed by PNP equations:

1 $$-{\varepsilon }_{0}{\varepsilon }_{r}{\nabla }^{2}\psi =F\sum {z}_{i}{c}_{i}$$

2 $$\nabla \cdot {\overrightarrow{N}}_{i}=\nabla \cdot (-{D}_{i}\nabla {c}_{i}-{z}_{i}\frac{{D}_{i}}{RT}F{c}_{i}\nabla \psi )=0$$

In the above equations; ψ is the electric potential, *F* is the Faraday constant ${\overrightarrow{N}}_{i}$ is the flux density, *D*_*i*_ is the diffusivity and R is the universal gas constant. One should bear in mind that PNP equations treats the ions as point charges, so ion size effects and dispersion forces between the ion-ion and ion-wall couplings are not considered in the literature^54^.

In order to include charge regulation nature of silica surface into the current model, the following dissociation/association reactions occurring at the solid/liquid interface are taken into account. The reactions in our system are assumed to be instantaneous, since the Damkohler numbers greater than 1 were calculated for the current system (Da»1) suggesting much faster reaction rates compared to the ionic transport.

3 $$SiOH\leftrightarrow Si{O}^{-}+{H}^{+}$$

4 $$SiOH+{H}^{+}\leftrightarrow SiO{H}_{2}^{+}$$

The equilibrium constants of these reactions can be calculated as:

5 $${K}_{A}=\frac{{{\rm{\Gamma }}}_{Si{O}^{-}}{[{H}^{+}]}_{w}}{{{\rm{\Gamma }}}_{SiOH}},\,{K}_{B}=\frac{{{\rm{\Gamma }}}_{SiO{H}_{2}^{+}}}{{{\rm{\Gamma }}}_{SiOH}{[{H}^{+}]}_{w}}$$

Here; ${{\rm{\Gamma }}}_{{{\rm{SiO}}}^{-}}$, ${{\rm{\Gamma }}}_{SiOH}$ and ${{\rm{\Gamma }}}_{SiO{H}_{2}^{+}}$ are the surface site densities of corresponding functional groups and [*H*^+^]_*w*_ is the concentration of hydrogen ion at the interface. Finally, surface charge density can be evaluated as:

6 $${\sigma }_{w}=-\frac{F{{\rm{\Gamma }}}_{total}}{{N}_{A}}\frac{{K}_{A}-{K}_{B}{{[{H}^{+}]}_{w}}^{2}}{{K}_{A}+{[{H}^{+}]}_{w}+{K}_{B}{{[{H}^{+}]}_{w}}^{2}}$$

We solved PNP equations in 2-D cartesian coordinates using Finite Elements Method. In the simulations: following values are used for the parameters: ε_0_ε_r_ = 7.08 × 10^−10^ F/m, R = 8.31 J/(mol·K), F = 96485 C/mol, *T* = 300 K, N_total_ = 4.816 1/nm^2^, pK_A_ = −logK_A_ = 7, and pK_B_ = −logK_B_ = 1.9. The diffusivities of H^+^, K^+^, Cl^−^ and OH^−^ ions are taken as 9.31 × 10^−9^, 1.957 × 10^−9^, 2.032 × 10^−9^ and 5.3 × 10^−9^ m^2^/s, respectively. Current model was validated with the approximate analytical solution for a semi-infinite flat surface theory^55^. Potential distributions calculated by iterations until an equilibrium forms and potential measured at internal center lines becomes equal to potential assigned at outer center lines.

**Figure 1:**
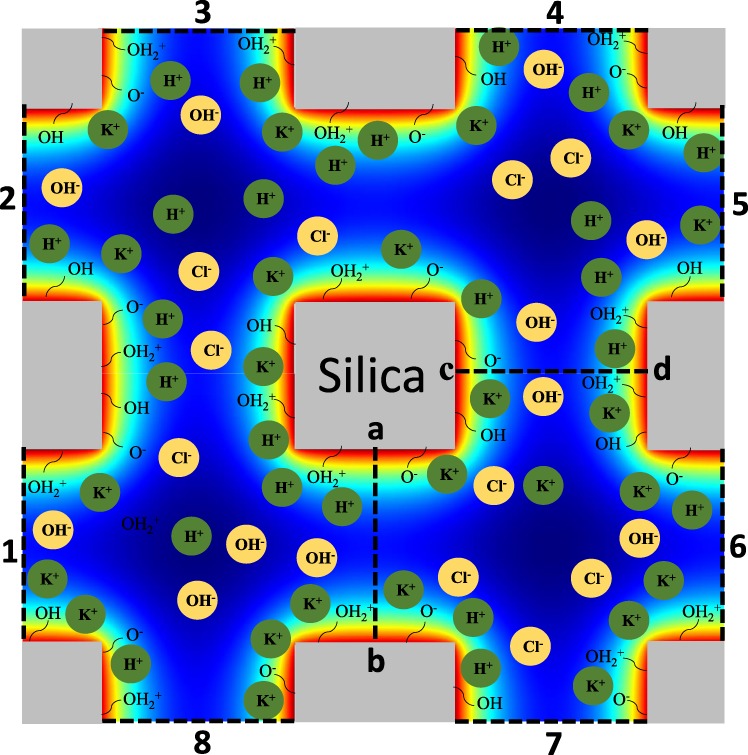
Schematic illustration of the solution domain consists of silica surfaces and four different ionic species.

Current study focuses on a representative volume of a mesoporous silica structure shown in Fig. 1. Potential distributions calculated by iterations. First, bulk conditions were assigned to ionic boundaries labeled by numbers 1 to 8 in Fig. 1 at first iteration. Then, the electric potential and ionic concentration distributions developed through the center lines denoted as “ab” and “cd” were measured and used as on out boundaries of 1 to 8 for the next iteration. This procedure continued until the equilibrium forms and potential measured at internal center lines becomes equal to potential assigned at outer center lines.

## Results and Discussion

**Figure 2:**
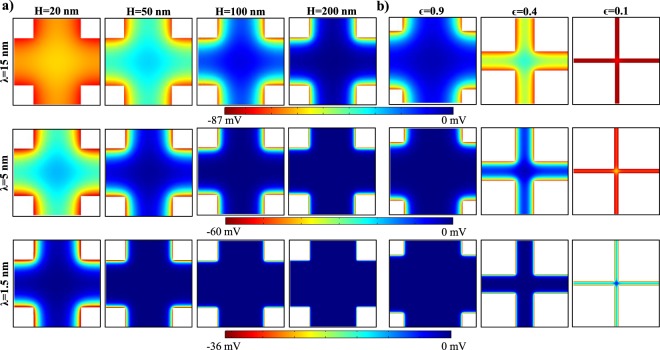
(**a**) Electric potential contours of varying EDL thickness and pore size at the constant porosity of 0.8. (**b**) Electric potential contours of varying EDL thickness and porosity at the constant pore size of 100 nm.

Mesoporous network was defined by pore size (H) and porosity (ϵ) while the aspect ratio of solid parts was kept unity. The ionic liquid was defined by the salt concentration and pH, as a function of which we calculated the thickness of resulted EDL (λ). Hence, we tried to investigate the nanoscale overlap effects as a function of the length scales characterizing the ionic liquid inside the nanopores, which are H, ϵ and λ. As a first step, we studied the electric potential distributions inside the mesopores of different pore sizes (H = 20 nm, 50 nm, 100 nm and 200 nm) with different EDL thicknesses (λ = 1.5 nm, 5 nm and 15 nm) at a constant porosity of ϵ = 0.8 and pH = 6. The resulting electric potential contours of each case is presented in Fig. 2a where each row is at the same salt concentration and each column is at the same pore size. First, when the EDL thickness is thin and/or pore size is big, zero electric potential develops at the center where negative and positive ions are equal to each other. We observed this for all pores sizes of 20–200 nm studied for λ = 1.5 nm and all studied salt concentrations of λ = 1.5–15 nm for H = 200 nm. However, with a decrease in salt concentration increasing the thickness of EDL or a decrease in pore size, EDLs grew from facing pore surfaces overlapped and the bulk potential became different than zero. These are the cases where the EDL thickness becomes non-negligible compared to pore size. Additionally, non-homogenous potential distribution developed on the pore surfaces due to the interaction/overlap of potential at the pore throats with the potential at the pore volume. Hence, pore to throat connectivity affects the ionic potential by decreasing H and λ.

**Figure 3:**
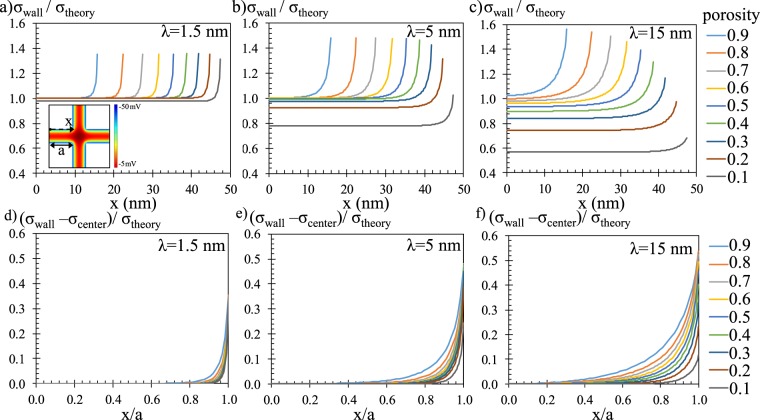
Surface charge density distribution on pore surface normalized with flat surface theory at different porosities and at different EDL thicknesses of (**a**) λ = 1.5 nm, (**b**) λ = 5 nm, and (**c**) λ = 15 nm for pore size of H = 100 nm. (**d**–**f**) Same charge distributions along axial direction normalized with pore throat length (l_t_) when the corresponding center equilibrium charge values were subtracted from the local charge variation.

Next, we studied the porosity values of 0.9, 0.4 and 0.1 at the EDL thicknesses of 1.5 nm, 5 nm and 15 nm for the constant pore size of 100 nm. Figure 2b presents the electric potential contours. Decreasing porosity resulted in narrower but longer throats in the system promoting EDL overlap and reduced the effect of pore throat to pore volume interactions. We further calculated and plotted the surface charge density along the pore surface of these cases in Fig. 3a–c. Surface charges were normalized with the flat surface theory in order to compare them. Overall, surface charge remained constant away from the corners of pore connection where potential interactions in the axial direction creates local variation. These local variation’s extents into pore throats were different depending on the pore throat length and EDL thickness. We described corresponding charging mechanisms in our earlier study^46^ where an electrokinetic development length of surface charging for short nano-conduits was defined and discussed in detail. Simply, there are two major charging mechanisms in a pore; an increase of EDL overlap decreases the normalized charge density while the pore throat to pore volume connectivity increases the normalized charge. The effect of EDL overlap results in as high as 40% decrease in normalized charge and pore connectivity creates up to 60% increase in local charge values. Next, we characterized these effects by normalizing axial direction by the corresponding pore surface length (l_t_) in Fig. 3d–f. In order to distinguish the effect of EDL overlap from pore connectivity, we subtracted the charge density value equilibrated at the center of pore throat from the charge distribution along the pore surfaces. Pore connectivity became effective with the increase of the EDL thickness, which lines with the conclusion of our earlier study where development length was found as 2.7 × λ. Porosity has negligible effects for non-overlapping case; local variation remained constant while its comparable coverage slightly decreased by the decrease of porosity due to the increasing l_t_. However, once the overlap starts, an increase in porosity increases the overlap effects which represses the local variation and increment of surface charge due to pore connectivity effects. For example, in the case of high connectivity effects at λ = 15 nm, increased porosity regressed the local charge increase and variation back to low effects cases of λ = 1,5 nm and 5 nm.

**Figure 4:**
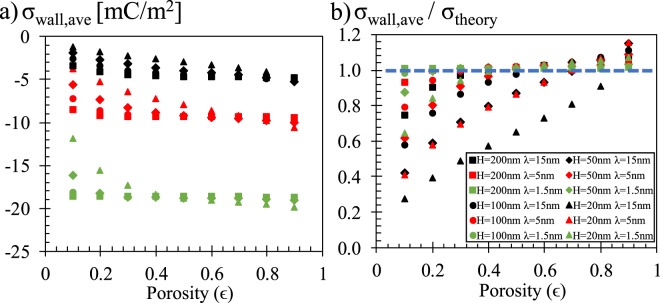
(**a**) Average charge on pore surface as a function of porosity at different pore sizes and EDL thicknesses. (**b**) Same results when the average surface charge is normalized by the theoretical calculations.

Next, we calculated an overall charge value as the average of charge distribution on surface for all cases of different pore sizes, EDL thicknesses and porosities. The average surface charge values as a function of porosity are presented in Fig. 4a. Silica surface developed negative surface charge which increased by the increase in salt concentration. Overall, absolute value of average surface charge increases with increasing porosity; but this effect is more prominent at small pore sizes. The absolute average charges of small pore size cases were higher at low porosities while absolute average charges of small pore sizes became equal to, and later lower than the higher pore size cases by the increased porosity. In Fig. 4b, average surface charges normalized by the flat surface theory were plotted. In the case of high EDL thickness, small pore size and low porosity, mesoporous surface charge decreased as low as 20% of the theoretical predictions. Deviation from theory was mostly observed for low porosities while high porosity values develops average charges higher than theory.

**Figure 5:**
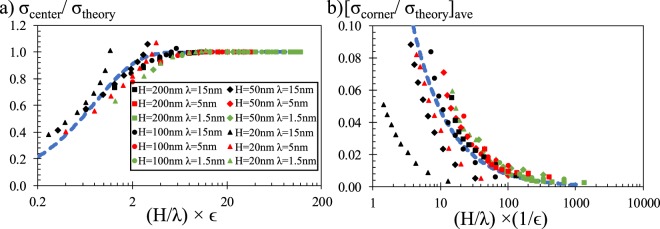
(**a**) Center charge and (**b**) average of the remaining charge distribution through pore surface as a function of non-dimensional groups of pore size, EDL thickness and porosity.

In order to characterize the behavior observed in Fig. 4b, we tried to distinguish the effects of overlap and connectivity. In a porous system, both effects develop as a function of the ratio between pore size and EDL thickness. We need to note here that the classical representation of a 2D porous system defines a representative volume with identical size in both dimensions; pore height and length are equal to each other. For example, when the throat height is comparable with EDL thickness, EDLs from opposing pore surfaces overlap; or, when throat lengths are comparable with EDL thickness, potential interaction through pore network takes place in more of the pore throat. Therefore, H/λ can describe both effects. However, effect of porosity develops differently. Overlap increases with decreasing porosity while connectivity effect increases with increasing porosity. For such a case, we studied equilibrium pore charge developing at throat center as a function of (H/λ) × ϵ in Fig. 5a and the average of the remaining charge distribution after subtracting the equilibrium center value as a function of (H/λ) × (1/ϵ) in Fig. 5b. Results from more than 100 different cases showed a very general distribution in both figures. Pore center surface charge remained the same with theoretical predictions until the (H/λ) × ϵ became smaller than 8, after which absolute value of center charge diverge from theory and decreased exponentially by decreasing (H/λ) × ϵ. Next, pore connectivity effects were mostly negligible for (H/λ) × (1/ϵ) values higher than 100 while decrease of (H/λ) × (1/ϵ) below 100 increased the effects of connectivity. Almost all of the data in Fig. 5b line up showing a similar variation. However, when the pore length became shorter than the electrokinetic development length, surface charge inside the pore throat cannot reach its equilibrium at the center section. We can characterize this behavior by the ratio of pore size to EDL thickness. As previously mentioned, our earlier study on short channels presented a 2.7 × λ long development length at which surface charge reaches %99 of its equilibrium value; however, surface charge reached 90% of its equilibrium value at approximately ~λ axial length of this slowly converging charge variation. The variation of connectivity effects showed a very similar profile as a function of (H/λ) × (1/ϵ) until H/λ becomes ~5 and smaller (H/λ = 5 case has pore throat center to pore volume distance of <2.5 × λ). Differentiation was still negligible for H/λ < 2 that only the results of H = 20 nm with λ = 15 nm case behaves different than the general variation.

Next, we developed an empirical model to fit our numerical results in an attempt to provide an easy to use phenomenological model describing mesoporous surface charge behavior. Variation of normalized charge at throat’s center presented an exponential behavior between two asymptotes of zero and one, as a function of the defined non-dimensional group of (H/λ) × ϵ. On the other hand, average of remaining charge distribution normalized with theory increased as an inverse power of non-dimensional group of (H/λ) × (1/ϵ). The mathematical fits are given in Fig. 5a,b as dashed blue lines. A combination of these two models given in Equation (7) offers a very simple set of equations characterizing mesoporous silica pore surface charging as a function of pore size, EDL thickness and porosity.

7 $${[\frac{\sigma }{{\sigma }_{theory}}]}_{ave}=1-\exp (-\frac{H\times \varepsilon }{\lambda })+0.3{(\frac{H}{\lambda \times \varepsilon })}^{-0.8}$$

**Figure 6:**
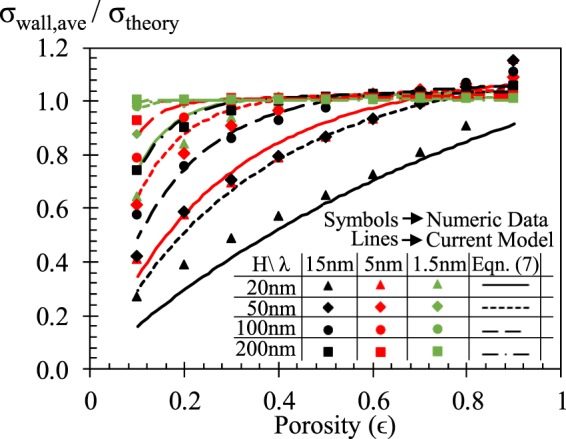
Comparison between predictions of new model (Eqn (7)) and numerical calculations.

The new model is tested on numerical results in Fig. 6. The solid symbols represent the simulation results, while the solid lines with empty markers are the calculations of Equation (7). The current model can predict the average surface charge density of mesoporous silica successfully at various condition.

## Conclusion

Mesoporous silica is becoming a practical material for many of the revolutionary nano-technological applications. Current experiments showed that internal surface charging of these nanoscale porous systems are different than the predictions of existing surface charge calculations. However, none of these experimental studies described and/or characterized the surface charge mechanisms inside mesoporous silica systems. To accomplish this, we examined divergence of internal surface charge from existing theory by varying pore size, porosity and ionic concentration. We observed that overlap of the EDLs from opposite surfaces of pore throats decreases the surface charge as a function of overlap ratio (H/λ). On the other hand, ionic diffusion through the pore connectivity between pore throat and pore volume increases surface charge, while this increase decreases and surface charge converges to its equilibrium value corresponding EDL overlap at a distance we defined as “the electrokinetic development length” in the axial direction. From our pore level analysis, we calculated an averaged surface charge on which we characterized the effects of overlap and connectivity separately. For the current representative volume of a porous system with unity aspect ratio, both mechanisms governed by the height and length of porous system developed as a function of H/λ. Hence, we achieved to characterize the variation of internal surface charge in terms of overlap ratio and porosity. We applied curve fitting using mathematical functions which suits physical behavior best in all extent of corresponding parameters’ range. As a result, we developed a phenomenological model which can predict the average mesoporous internal surface charge as a function of EDL thickness, pore size and porosity for the first time in literature.

## Electronic supplementary material

SI

## Data Availability

The datasets generated during the current study are available from the corresponding author on reasonable request.
