# Supplementary material for: Internal surface electric charge characterization of mesoporous silica
Source: Sci Rep. 2019 Jan 15;9:137. doi: 10.1038/s41598-018-36487-w (PMC6333811; doi:10.1038/s41598-018-36487-w)
Supplement: Supplementary file 1 — SI [file 41598_2018_36487_MOESM1_ESM.pdf]

Supporting Information

Internal surface electric charge characterization of mesoporous silica

Tumcan Sen[1], Murat Barisik[1*]

[1]Department of Mechanical Engineering, Izmir Institute of Technology, IZMIR, 35430

* Corresponding author E-mail: muratbarisik@iyte.edu.tr

Simulation Details

Current model was validated with the approximate analytical solution for a semi-infinite flat surface theory.[S1] To satisfy semi-infinite surface condition, high KCl concentrations (i.e. 10 mM and 100 mM) are used in the simulations for various pH values. In these high concentration cases, EDL fields do not overlap and the resulting surface charge density values can be described by the solution of PB. Figure S1 shows the surface charge density values for pH range from 3 to 9. The increasing trend of surface charge density with pH and KCl concentration can easily be distinguished in the figure. The current model (markers) yields identical results with the theory (solid lines).

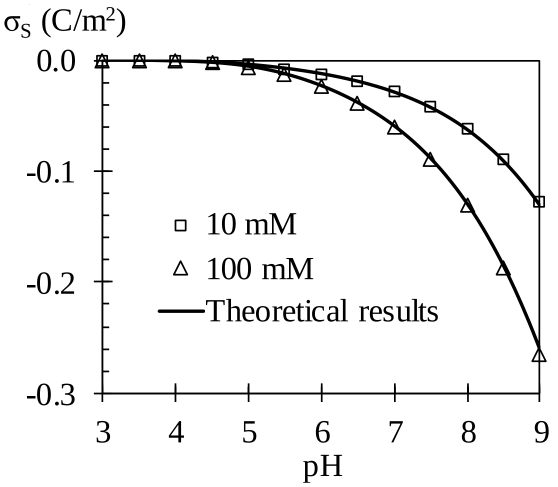

Figure S1. Surface charge densities inside mesoporous silica. Lines indicate the theoretical results while the markers are the results of the numerical solution.

S1. Yeh, L.-H., Xue, S., Joo, S. W., Qian, S. & Hsu, J.-P. Field Effect Control of Surface Charge Property and Electroosmotic Flow in Nanofluidics. *The Journal of Physical Chemistry C* **116**, 4209-4216 (2012).
